# Analysis of factors influencing hemoglobin target achievement in maintenance hemodialysis patients in Wuhan, China

**DOI:** 10.3389/fmed.2025.1665787

**Published:** 2026-01-26

**Authors:** Jingyi Liu, Yonglong Min, Nan Jiang, Hong Liu, Xiaohui Wang, Fei Xiong

**Affiliations:** 1 Department of Nephrology, The Fifth Hospital of Wuhan, Wuhan, China; 2 Department of Nephrology, Wuhan First Hospital, Wuhan, China

**Keywords:** maintenance hemodialysis, hemoglobin target achievement, anemia management, influencing factors, clinical variables

## Abstract

**Introduction:**

This study identified key factors influencing hemoglobin (Hb) target achievement (≥110 g/L) in maintenance hemodialysis (MHD) patients in Wuhan to guide improvement strategies.

**Methods:**

A multicenter retrospective study (January 2019–December 2023) included 4,906 MHD patients from 70 dialysis centers in Wuhan. Data on demographics, dialysis-related parameters, pre-dialysis laboratory indices, and medication use were collected. Patients were categorized into target (Hb ≥ 110 g/L) and non-target group (Hb < 110 g/L). Group differences were analyzed, and binary multivariate logistic regression identified independent factors.

**Results:**

A total of 4,906 MHD patients from 70 dialysis centers were included in this study. Multivariate logistic regression analysis identified female sex (*OR* = 1.18, *p* = 0.011), TCC use (*OR* = 1.23, *p* = 0.006), dialysis frequency <3 times/week (*OR* = 1.20, *p* = 0.006), pre-dialysis hypertension (*OR* = 1.28, *p* < 0.001), lack of LC use (*OR* = 1.16, *p* = 0.043), higher serum phosphorus (pre 0.1 mmol/L *OR* = 1.02, *p* < 0.001) and CRP (pre 1 mg/dL *OR* = 1.01, *p* < 0.001) as independent risk factors for not achievement. Independent protective factors included LMWH use (*OR* = 0.59, *p* = 0.002), coverage under employee medical insurance (*OR* = 0.74, *p* = 0.033), higher serum albumin (pre 1 g/L *OR* = 0.90, *p* < 0.001), calcium (per 0.1 mmol/L *OR* = 0.95, *p* < 0.001), and TSAT (pre 1% *OR* = 0.99, *p* < 0.001).

**Conclusion:**

Hb target achievement in Wuhan MHD patients is influenced by a complex interplay of demographic (sex), clinical (vascular access type, dialysis adequacy and frequency, blood pressure management, inflammatory status, mineral/nutrition/iron status), therapeutic (LMWH and LC management), and socioeconomic factors (insurance). Targeting modifiable factors is crucial for optimizing anemia management.

## 1 Introduction

Chronic kidney disease (CKD), marked by high prevalence, disability, and low awareness, has emerged as a major global public health challenge. According to the 2023 *ISN Global Kidney Health Atlas*, over 850 million people worldwide are affected by CKD, with a median prevalence of 9.5% (1). As the disease advances, many patients progress to end-stage renal disease (ESRD), necessitating renal replacement therapies such as hemodialysis (HD) or kidney transplantation. Data from the 2025 *Chinese Renal Data System (CNRDS)* show that by the end of 2024, the number of HD patients in mainland China had reached 1,027,267, with a prevalence exceeding 600 per million—representing a 4.3-fold increase since 2011. Renal anemia is among the most common complications in CKD, especially in maintenance hemodialysis (MHD) patients, with a reported prevalence exceeding 90% (2, 3). It significantly compromises quality of life (4) and is associated with increased risks of cardiovascular events, hospitalization, healthcare costs, and mortality (5, 6). In recent years, various international and national guidelines have recommended a target hemoglobin (Hb) level of 110–130 g/L for MHD patients, with an emphasis on individualized therapy to improve patient outcomes (7, 8). However, data from the Dialysis Outcomes and Practice Patterns Study (DOPPS) revealed that as of 2021, only 43.1% of Chinese MHD patients met the Hb target (9). To enhance dialysis quality, the CNRDS was launched in 2010, followed by the establishment of multiple provincial and municipal quality control platforms. In Wuhan, the Hemodialysis Quality Control Center was established in 2009, and a citywide digital platform was implemented in 2017. This system now covers 70 dialysis units, with regular reporting of MHD patient data, including epidemiological profiles, primary diseases, complications, outcomes, dialysis parameters, and quality control laboratory indices. Over the past years, real-time monitoring and data analysis via this platform have played a critical role in promoting the standardized development of HD care in Wuhan, particularly in improving Hb target attainment. Leveraging data from the Wuhan Hemodialysis Quality Control Platform between 2019 and 2023, this study aims to explore factors associated with Hb target achievement in MHD patients, providing data-driven evidence to support optimized anemia management strategies in clinical dialysis practice.

## 2 Materials and methods

### 2.1 Population

This retrospective study included patients receiving MHD at 70 dialysis centers in Wuhan, China, as registered in the Wuhan Hemodialysis Quality Control System^1^ between January 1, 2019, and December 31, 2023. The study protocol was approved by the Ethics Committee of Wuhan First Hospital (Approval No.: KL [2024]70). *Inclusion criteria*: Age ≥18 years; Diagnosed with ESRD and undergoing regular MHD; Registered in the Wuhan Hemodialysis Quality Control Platform; Provision of informed consent. *Exclusion criteria*: Coexisting conditions that could independently cause anemia, such as malignancies; gastrointestinal diseases (e.g., liver cirrhosis, peptic ulcers); hematological disorders (e.g., leukemia); or autoimmune/connective tissue diseases (e.g., systemic lupus erythematosus); Discontinuation of HD due to transfer to peritoneal dialysis or withdrawal from therapy; History of kidney transplantation; Acute kidney injury requiring temporary HD due to drugs, infections, or other reversible causes; Duration of MHD treatment less than 3 months; Incomplete or inaccurate clinical data.

### 2.2 Methods

#### 2.2.1 Data collection

Patient data from January 1, 2019, to December 31, 2023, were extracted from the Wuhan Hemodialysis Quality Control Center online platform. Collected variables included: *Demographics:* sex, age, primary disease, type of medical insurance; *Infectious status:* hepatitis B virus (HBV), hepatitis C virus (HCV), syphilis; *Dialysis-related variables:* dialysis vintage (months), vascular access type, pre-dialysis blood pressure, single-pool Kt/V (spKt/V), dialysis frequency, dialysis regimen, and anticoagulation type; *Medications:* use of erythropoietin(EPO), hypoxia-inducible factor prolyl hydroxylase inhibitors (HIF-PHIs), iron supplements, L-carnitine (LC), folic acid, vitamin B12, active vitamin D, calcimimetics, and phosphate binders; *Pre-dialysis laboratory indicators:* albumin (Alb), potassium (K), calcium (Ca), phosphorus (P), C-reactive protein (CRP), parathyroid hormone (PTH), serum ferritin (SF), and transferrin saturation (TSAT).

#### 2.2.2 Treatment protocol

As part of the standardized clinical practice across the participating centers, the following key treatment protocols were implemented.

The 70 participating centers encompassed tertiary hospitals in urban areas and secondary hospitals or clinics in suburban/rural regions. While all adhered to the unified quality control standards of the Wuhan Hemodialysis Quality Control Center, variations in facility resources (e.g., specialist availability, equipment) between settings may have influenced aspects of patient care.

*Hb Measurement*: Hemoglobin was measured using Mindray BC-series hematology analyzers with the matched M-68LH lytic reagent (colorimetric method). A citywide mutual recognition policy for laboratory results in Wuhan ensures that all participating centers adhere to this identical standardized protocol and quality control standards.

*Dialyzer*: All hemodialysis treatments were performed using single-use, high-flux dialyzers.

*Anticoagulation*: For patients prescribed with low-molecular-weight heparin (LMWH) as the anticoagulant, it was administered as an intravenous bolus of 60–80 anti-Xa IU per kilogram of body weight at the initiation of each hemodialysis session, in accordance with the Chinese Standard Operating Procedures for Blood Purification (2021 Edition).

*EPO*: Recombinant human erythropoietin (rHuEPO) was administered intravenously at the end of dialysis, at a dosage of 50–150 IU/kg per week, 1–3 times a week.

*Iron Supplements*: Intravenous iron was administered as a total cumulative dose of 1,000 mg per course, with a maintenance dose of 100 mg every 1–2 weeks after iron repletion. The oral iron supplement dose was 200 mg of elemental iron per day.

*LC*: L-carnitine was supplemented intravenously at a dose of 1 g (one vial of 5 mL: 1 g) after each dialysis session.

#### 2.2.3 Diagnostic criteria

According to the Chinese Clinical Practice Guidelines for the Diagnosis and Management of Renal Anemia, the target Hb level for renal anemia management in MHD patients is ≥110 g/L, applicable to both males and females. In reference to the Chinese Guidelines for the Management of Hypertension in CKD Patients (2023 Edition), pre-dialysis blood pressure should be <140/90 mmHg in MHD patients younger than 60 years.

#### 2.2.4 Patient grouping

Based on the guidelines, patients were categorized into two groups according to their last pre-dialysis Hb measurement (as of December 31, 2023): Target Hb group: Hb ≥ 110 g/L; Non-target Hb group: Hb < 110 g/L.

### 2.3 Statistical analysis

Statistical analysis was performed using SPSS version 27.0. Normality of continuous variables was assessed using the Kolmogorov–Smirnov test. Normally or approximately normally distributed data were expressed as mean ± standard deviation (*x ± s*), while non-normally distributed data were presented as median and interquartile range [*M (P25, P75)*]. Between-group comparisons were conducted using independent sample t-tests or Mann–Whitney U tests, as appropriate. Categorical variables were presented as counts and percentages [*n* (%)], and compared using the Chi-square (*χ^2^*) test. Variables with statistically significant differences (*p* < 0.05) in univariate analysis were included as independent variables in a multivariate binary logistic regression model, with Hb target achievement (yes/no) as the dependent variable, to identify independent predictors of target Hb attainment.

## 3 Results

### 3.1 Comparison of baseline characteristics

A total of 4,906 MHD patients were included in this study based on predefined inclusion and exclusion criteria. Among them, 2,906 patients achieved the Hb target, while 2,000 did not. The overall cohort was predominantly male (63.0%, 3,092/4,906), with a mean age of 58.55 ± 13.98 years and a median dialysis vintage of 36 months. The largest proportion of patients initiated dialysis between the ages of 60 and 74 years (39.5%, 1,938/4,906). Comparative analysis revealed that the Hb target group had a higher percentage of males (65.0% vs. 60.2%) and a longer median dialysis duration [38 (19, 71) vs. 33 (15, 61.75) months] than the non-target group. Conversely, the mean age at dialysis initiation was lower in the target-achieved group compared to the non-target group (57.51 ± 13.82 vs. 60.07 ± 14.06 years). All differences reached statistical significance (*p* < 0.001).

Primary glomerulonephritis (32.0%, 1,569/4,906), diabetic nephropathy (27.2%), and hypertensive nephropathy (21.5%) were the leading causes of ESRD, with no significant differences in their distribution between groups (*p* = 0.067). The prevalence of infectious diseases was relatively low, including hepatitis B (5.7%), hepatitis C (1.5%), and syphilis (1.2%), with no significant intergroup differences observed (*p* > 0.05). Regarding insurance status, urban employee medical insurance accounted for the largest proportion (17.5%, 860/4,906). Notably, the proportion of patients covered by basic medical insurance for urban and rural residents was significantly lower in the Hb target group compared to the non-target group (5.3% vs. 7.2%) (*p* = 0.006), whereas the difference in the proportion of urban employee insurance patients was not significant (17.8% vs. 17.4%) (*p* = 0.736). The independent association of insurance type with Hb achievement was further assessed in the multivariate model (Table 1).

**Table 1:** Comparison of baseline characteristics between the two groups.

Variable	Hb target group (*n* = 2,906)	Hb non-target group (*n* = 2,000)	Test statistic (*t/z/χ^2^*)	*p*-value
Male, *n* (%)	1,888 (65.0%)	1,204 (60.2%)	11.562	<0.001
Age at dialysis initiation (years)	57.51 ± 13.82	60.07 ± 14.06	6.311	<0.001
Age group at initiation, *n* (%)			39.856	<0.001
18–44	533 (18.3%)	308 (15.4%)	7.216	0.007
45–59	967 (33.3%)	575 (28.7%)	11.260	<0.001
60–74	1,118 (38.5%)	820 (41.0%)	3.168	0.075
≥75	288 (9.9%)	297 (14.9%)	27.552	<0.001
Dialysis vintage (months), median (IQR)	38 (19, 71)	33 (15, 61.75)	−5.246	<0.001
Primary renal disease, *n* (%)				
Primary glomerulonephritis	976 (33.6%)	593 (29.7%)	8.782	0.067
Diabetic nephropathy	767 (26.4%)	568 (28.4%)		
Hypertensive nephropathy	616 (21.2%)	437 (21.8%)		
Others	276 (9.5%)	206 (10.3%)		
Unknown	271 (9.3%)	196 (9.8%)		
Infectious diseases				
Hepatitis B virus infection, *n* (%)	163 (5.6%)	116 (5.8%)	0.081	0.777
Hepatitis C virus infection, *n* (%)	50 (1.7%)	23 (1.2%)	2.631	0.105
Syphilis, *n* (%)	38 (1.3%)	23 (1.2%)	0.240	0.624
Insurance type, *n* (%)			7.940	0.019
Basic medical insurance for urban and rural residents	154 (5.3%)	144 (7.2%)	7.501	0.006
Urban employee basic medical insurance	505 (17.4%)	355 (17.8%)	0.114	0.736
Others/unknown	2,247 (77.3%)	1,501 (75.0%)	3.394	0.065

### 3.2 Comparison of dialysis-related parameters

Analysis of dialysis vascular access revealed that the use of autologous arteriovenous fistulas (AVFs) was significantly more common in the Hb target group than in the non-target group (74.9% vs. 65.2%, *p* < 0.001). In contrast, tunneled cuffed catheters (TCCs) and non-cuffed, non-tunneled catheters (NCCs) were used less frequently in the target group (TCC: 21.5% vs. 30.4%, *p* < 0.001; NCC: 0.9% vs. 1.7%, *p* = 0.017). Both groups primarily received dialysis three or more times per week; however, this frequency was more prevalent in the target group (65.9% vs. 59.7%, *p* < 0.001). Pre-dialysis blood pressure control was also more frequently achieved in the target group (55.9% vs. 50.3%, *p* < 0.001). Dialysis adequacy, defined as spKt/V ≥ 1.2, was significantly higher in the Hb target group (80.5% vs. 77.8%, *p* = 0.018). There were no significant differences in dialysis modalities between groups (*p* = 0.333), with conventional HD being the most common approach in both. According to the Chinese Standard Operating Procedures for Blood Purification (2021 Edition), LMWH was administered as an intravenous bolus (60–80 anti-Xa IU/Kg) at the start of each hemodialysis session, which is a standard and ethical practice for hemodialysis anticoagulation. Low-molecular-weight heparin (LMWH) was the predominant anticoagulant used across both groups, with a significantly higher usage rate in the target group (83.7% vs. 79.5%, *p* < 0.001) (Table 2).

**Table 2:** Comparison of dialysis-related parameters between Hb target and non-target groups.

Variable	Hb target group (*n* = 2,906)	Hb non-target group (*n* = 2,000)	Test statistic (*t/z/χ^2^*)	*p*-value
Vascular access			66.661	<0.001
AVF	2,176 (74.9%)	1,304 (65.2%)	53.835	<0.001
TCC	626 (21.5%)	609 (30.4%)	49.911	<0.001
NCC	26 (0.9%)	33 (1.7%)	5.688	0.017
AVG	59 (2.0%)	28 (1.4%)	2.702	0.100
Others	19 (0.7%)	26 (1.3%)	5.443	0.020
Dialysis frequency			20.145	<0.001
≥3 times/week	1,916 (65.9%)	1,193 (59.7%)		
<3 times/week	990 (34.1%)	807 (40.3%)		
Pre-dialysis blood pressure			14.855	<0.001
<140/90 mmHg	1,624 (55.9%)	1,006 (50.3%)		
≥140/90 mmHg	1,282 (44.1%)	994 (49.7%)		
Dialysis adequacy (spKt/V)			5.568	0.018
≥1.2	2,340 (80.5%)	1,555 (77.8%)		
<1.2	566 (19.5%)	445 (22.2%)		
Dialysis modality			4.583	0.333
HD	2,027 (69.8%)	1,436 (71.8%)		
HDF	21 (0.7%)	21 (1.0%)		
HD + HDF	768 (26.4%)	484 (24.2%)		
HD + HP	25 (0.9%)	16 (0.8%)		
HD + HDF + HP	65 (2.2%)	43 (2.2%)		
Anticoagulant type			19.207	<0.001
UFH	84 (2.9%)	83 (4.1%)	5.991	0.014
LMWH	2,433 (83.7%)	1,590 (79.5%)	14.317	<0.001
Citrate	12 (0.4%)	20 (1.0%)	6.301	0.012
No anticoagulant	10 (0.3%)	9 (0.5%)	0.344	0.557
Others	367 (12.7%)	298 (14.9%)	5.214	0.022

### 3.3 Comparison of medication usage

Medication analysis showed that patients in the Hb target group had significantly higher usage rates of iron supplements (69.2% vs. 65.8%, *p* = 0.012), levocarnitine (LC) (48.5% vs. 45.1%, *p* = 0.018), active vitamin D (58.1% vs. 52.4%, *p* < 0.001), and phosphate binders (65.7% vs. 55.2%, *p* < 0.001) compared to the non-target group. No significant differences were observed between the two groups in the use of erythropoiesis-stimulating agents (ESAs; primarily EPO) (94.9% vs. 95.1%), hypoxia-inducible factor prolyl hydroxylase inhibitors (HIF-PHIs) (1.9% vs. 2.5%), folic acid (4.6% vs. 5.7%), vitamin B12 (4.0% vs. 3.3%), or calcimimetics (3.0% vs. 2.4%) (all *p* > 0.05) (Table 3).

**Table 3:** Comparison of medication usage between Hb target and non-target groups.

Medication	Hb target group (*n* = 2,906)	Hb non-target group (*n* = 2,000)	Test statistic (*χ^2^/t/z*)	*p*-value
Erythropoiesis-stimulating agents (ESAs)	2,758 (94.9%)	1,901 (95.1%)	0.051	0.822
Iron supplements	2,011 (69.2%)	1,316 (65.8%)	6.281	0.012
HIF-PH inhibitors (HIF-PHIs)	55 (1.9%)	49 (2.5%)	1.774	0.183
Levocarnitine (LC)	1,410 (48.5%)	902 (45.1%)	5.562	0.018
Folic acid	135 (4.6%)	113 (5.7%)	2.490	0.115
Vitamin B12	116 (4.0%)	65 (3.3%)	1.834	0.176
Active vitamin D	1,688 (58.1%)	1,047 (52.4%)	15.804	<0.001
Calcimimetics	86 (3.0%)	48 (2.4%)	1.395	0.238
Phosphate binders	1,910 (65.7%)	1,103 (55.2%)	55.918	<0.001

### 3.4 Comparison of biochemical parameters

The Hb target group demonstrated significantly higher levels of serum albumin (39.6 vs. 37.7 g/L, *p* < 0.001), serum potassium (4.75 ± 0.79 vs. 4.66 ± 1.21 mmol/L, *p* = 0.002), serum calcium (2.25 ± 0.24 vs. 2.19 ± 0.23 mmol/L, *p* < 0.001), and transferrin saturation (27.93% vs. 24.49%, *p* < 0.001) compared with the non-target group. In contrast, serum phosphorus (1.64 ± 0.70 vs. 1.72 ± 0.71 mmol/L, *p* < 0.001) and C-reactive protein (0.5 vs. 0.86 mg/dL, *p* < 0.001) levels were significantly lower in the target group. Additionally, the median parathyroid hormone (PTH) level was slightly higher in the target group than in the non-target group (272.1 vs. 245.0 pg./mL, *p* = 0.027), whereas no significant difference was observed in serum ferritin levels between the two groups (127.85 vs. 150.0 μg/L, *p* = 0.090) (Table 4).

**Table 4:** Comparison of biochemical parameters between Hb target and non-target groups.

Parameter	Hb target group (*n* = 2,906)	Hb non-target group (*n* = 2,000)	Test statistic (*t/z*)	*p*-value
Albumin (g/L)	39.6 (37.3, 41.8)	37.7 (34.8, 40.2)	−16.877	<0.001
Potassium (mmol/L)	4.75 ± 0.79	4.66 ± 1.21	3.083	0.002
Calcium (mmol/L)	2.25 ± 0.24	2.19 ± 0.23	7.994	<0.001
Phosphorus (mmol/L)	1.64 ± 0.70	1.72 ± 0.71	−3.616	<0.001
C-reactive protein (mg/dL)	0.5 (0.21, 1.76)	0.86 (0.3, 4.22)	−9.863	<0.001
Parathyroid hormone (pg/mL)	272.1 (145.5, 469.96)	245.0 (134.29, 450.78)	−2.207	0.027
Ferritin (μg/L)	127.85 (52.03, 272.25)	150.0 (53.00, 308.60)	−1.694	0.090
Transferrin saturation (%)	27.93 (20.39, 37.70)	24.49 (16.47, 34.20)	−9.112	<0.001

### 3.5 Factors associated with hemoglobin target achievement

Variables with statistical significance in univariate analysis were entered into a multivariate binary logistic regression model. Independent variables included sex, age at dialysis initiation, dialysis vintage, vascular access type, dialysis frequency, pre-dialysis blood pressure control, dialysis adequacy (spKt/V), anticoagulant type, use of iron supplements, levocarnitine, active vitamin D, phosphate binders, serum albumin, potassium, calcium, phosphorus, C-reactive protein, parathyroid hormone, transferrin saturation, and medical insurance type. The dependent variable was achievement of the Hb target. The analysis revealed that female sex (*OR* = 1.18, 95% *CI*: 1.040–1.347), use of tunneled central venous catheter (TCC) (*OR* = 1.229, 95% *CI*: 1.062–1.424), dialysis frequency less than three times per week (*OR* = 1.195, 95% *CI*: 1.053–1.356), uncontrolled pre-dialysis blood pressure (*OR* = 1.275, 95% *CI*: 1.129–1.440), and absence of levocarnitine treatment (*OR* = 1.164, 95% *CI*: 1.005–1.347) were independently associated with increased risk of failing to reach Hb targets (all *p* < 0.05). Conversely, use of low-molecular-weight heparin (LMWH) (*OR* = 0.587, 95% *CI*: 0.422–0.816) and enrollment in urban employee medical insurance (*OR* = 0.736, 95% *CI*: 0.555–0.979) were protective factors, significantly reducing the risk of Hb target failure (both *p* < 0.05). Furthermore, each 1 g/L increase in serum albumin was associated with a 10.1% reduction in risk (*OR* = 0.899, 95% *CI*: 0.884–0.913, *p* < 0.001), and each 0.1 mmol/L increase in serum calcium reduced risk by 4.8% (*OR* = 0.952, 95% *CI*: 0.926–0.978, *p* < 0.001). In contrast, each 0.1 mmol/L increase in serum phosphorus increased risk by 1.7% (*OR* = 1.017, 95% *CI*: 1.007–1.027, *p* < 0.001), and each 1 mg/dL increase in C-reactive protein increased risk by 1.4% (*OR* = 1.014, 95% *CI*: 1.007–1.021, *p* < 0.001). Additionally, each 1% increase in transferrin saturation reduced the risk by 1% (*OR* = 0.990, 95% *CI*: 0.986–0.994, *p* < 0.001) (Table 5).

**Table 5:** Binary logistic regression analysis of factors associated with hemoglobin target achievement.

Variable	*β*	SE	Wald	*p*-value	*OR*	95% *CI*
Sex (female)	0.168	0.066	6.500	0.011	1.183	1.040–1.347
Age at dialysis initiation	−0.002	0.003	0.444	0.505	0.998	0.993–1.003
Dialysis vintage (months)	−0.001	0.001	1.828	0.176	0.999	0.997–1.000
Vascular access type			16.616	0.002		
AVF (reference)	–	–	–	–	1	–
TCC	0.207	0.075	7.620	0.006	1.229	1.062–1.424
NCC	0.488	0.277	3.118	0.077	1.629	0.948–2.802
AVG	−0.283	0.241	1.382	0.240	0.754	0.470–1.208
Others	0.734	0.318	5.328	0.021	2.083	1.117–3.883
Dialysis frequency (<3/week)	0.178	0.065	7.587	0.006	1.195	1.053–1.356
Pre-dialysis BP ≥ 140/90 mmHg	0.243	0.062	15.315	<0.001	1.275	1.129–1.440
spKt/V < 1.2	0.086	0.079	1.188	0.276	1.089	0.934–1.271
Anticoagulant type			14.551	0.006		
UFH (reference)	–	–	–	–	1	–
LMWH	−0.533	0.168	10.040	0.002	0.587	0.422–0.816
Citrate	0.200	0.421	0.224	0.636	1.221	0.534–2.788
No anticoagulant	−0.490	0.510	0.925	0.336	0.612	0.225–1.664
Others	−0.407	0.184	4.878	0.027	0.666	0.464–0.955
Iron supplementation use	−0.044	0.070	0.393	0.531	0.957	0.833–1.098
Levocarnitine use	0.151	0.075	4.097	0.043	1.164	1.005–1.347
Active vitamin D use	−0.038	0.072	0.273	0.601	0.963	0.837–1.109
Phosphate binder use	0.109	0.067	2.593	0.107	1.115	0.977–1.272
Albumin (g/L)	−0.107	0.008	163.685	<0.001	0.899	0.884–0.913
Potassium (mmol/L)	−0.015	0.032	0.232	0.630	0.985	0.925–1.048
Calcium (per 0.1 mmol/L)	−0.049	0.014	12.180	<0.001	0.952	0.926–0.978
Phosphorus (per 0.1 mmol/L)	0.017	0.005	11.487	<0.001	1.017	1.007–1.027
C-reactive protein (mg/dL)	0.014	0.004	15.442	<0.001	1.014	1.007–1.021
Parathyroid hormone (pg/mL)	0.049	0.042	1.555	0.212	0.988	0.961–1.117
Transferrin saturation (%)	−0.010	0.002	26.041	<0.001	0.990	0.986–0.994
Medical insurance type			8.847	0.012		
Urban and rural residents	–	–	–	–	1	–
Urban employee	−0.307	0.144	4.540	0.033	0.736	0.555–0.979
Others	−0.380	0.129	8.643	0.003	0.684	0.531–0.881
Constant	5.333	0.546	95.423	<0.001	207.090	–

## 4 Discussion

This multicenter retrospective study analyzed MHD patients from 70 dialysis centers in Wuhan between 2019 and 2023, identifying a range of factors significantly associated with Hb target achievement (≥110 g/L). Hb control was influenced by demographic characteristics (e.g., sex), clinical variables (vascular access type, dialysis frequency, spKt/V, blood pressure control, inflammatory status [CRP], nutritional status [albumin], mineral metabolism [calcium and phosphate], and iron availability [TSAT]), pharmacological interventions (use of low molecular weight heparin and levocarnitine), and insurance type. These findings underscore the multifactorial nature of anemia management in MHD patients and highlight the need for individualized, integrated approaches. Moreover, the results demonstrate the value of leveraging a regional quality control platform to support precision management and data-driven policy decisions, offering a practical foundation for improving local Hb target rates and optimizing clinical outcomes.

Multivariate binary logistic regression analysis identified several independent predictors of Hb target attainment in MHD patients. Female patients had a significantly higher risk of failing to reach the Hb target compared to males (*OR* = 1.183), consistent with the findings of Shiferaw et al. (10), who reported similar sex-related differences in a meta-analysis involving 28 studies and 24,008 patients. While biological differences including lower androgen levels and prior iron loss may contribute, it is important to consider the role of age and menopausal status. The mean age of female subjects in our cohort was 59.30 ± 14.59 years, suggesting that a substantial proportion were likely postmenopausal. Therefore, the observed association may not be driven by sex per se, but rather by age-related hormonal changes and altered iron metabolism associated with menopause, which are known to impair erythropoiesis. Future studies specifically designed to collect menopausal status are needed to disentangle the effects of sex from those of menopause. Moreover, type of medical insurance was also significantly associated with Hb outcomes. While the univariate distribution (Table 1) showed a higher proportion of resident-insured patients in the non-target group, multivariate binary logistic regression analysis—which adjusts for key clinical and socioeconomic confounders—identified enrollment in urban employee medical insurance as an independent protective factor for achieving the Hb target (OR = 0.736, *p* = 0.033). This robust adjusted finding suggests that patients with employee insurance have a higher likelihood of Hb target achievement compared to those with resident insurance. This suggests that differences in reimbursement policies may affect treatment accessibility and adherence, ultimately influencing anemia management (11, 12).

Secondly, Vascular access type emerged as a significant predictor of Hb control. Compared with arteriovenous fistulas (AVFs), the use of tunneled (TCC) or non-tunneled central venous catheters (NCC) was associated with a higher risk of Hb non-attainment, consistent with findings reported by Li et al. (13). AVFs typically allow for higher blood flow rates and more efficient clearance of uremic toxins, which improves dialysis adequacy and reduces the inhibitory effects of uremia on erythropoiesis (14, 15). Furthermore, patients undergoing dialysis ≥3 times per week had significantly better Hb control than those dialyzed less frequently. More frequent dialysis enhances uremic toxin clearance, attenuates chronic microinflammation, reduces hepcidin levels, and improves responsiveness to erythropoiesis-stimulating agents (ESAs) (16, 17). Inadequate blood pressure control was another independent risk factor: pre-dialysis hypertension (≥140/90 mmHg) likely contributes to endothelial dysfunction and systemic inflammation via upregulation of pro-inflammatory cytokines such as TNF-*α* and IL-6, thereby impairing endogenous erythropoietin (EPO) production and ESA responsiveness (18). Additionally, oxidative stress induced by hypertension may further aggravate ESA hyporesponsiveness (19). The choice of anticoagulant also influenced anemia outcomes; LMWH use was a protective factor. Compared with unfractionated heparin (UFH), LMWH has a longer half-life and stronger anti-Xa activity, and is frequently used in stable patients, improving blood flow and dialysis efficiency while minimizing thrombus-related blood loss (20, 21).

Additionally, regarding dialysis medications, our study identified that patients not receiving levocarnitine (LC) had a higher risk of failing to achieve target Hb levels, consistent with previous reports (22, 23). Maruyama et al. demonstrated that intravenous LC administration can alleviate ESA resistance caused by LC deficiency in dialysis patients, thereby reducing ESA dosage requirements (24). Intravenous LC supplementation has also been shown to significantly improve Hb levels and iron metabolism markers in MHD patients (22). These findings underscore the clinical significance of LC supplementation in managing anemia among MHD patients.

In terms of laboratory indicators, serum albumin (Alb) was identified as a protective factor for achieving target Hb levels, with each 1 g/L increase in Alb associated with a 10.1% reduction in the risk of Hb non-attainment. Prior studies have linked higher nutritional risk indices with lower Hb levels in MHD patients (25), emphasizing the importance of early detection and correction of malnutrition to improve anemia outcomes. Our study reaffirms serum albumin as a strong protective factor for Hb target achievement. While we did not collect direct dietary protein intake data, current clinical guidelines provide clear recommendations to support our findings. The Kidney Disease: Improving Global Outcomes (KDIGO) guidelines suggest maintaining serum albumin levels above 4.0 g/dL (40 g/L) in dialysis patients, which aligns with our observation of a significant benefit with each 1 g/L increase in albumin (26). Regarding protein intake, KDOQI Clinical Practice guideline recommend a dietary protein intake of 1.0–1.2 g/kg/day for stable maintenance hemodialysis patients to ensure adequate nutrition for erythropoiesis and overall health (27). Therefore, optimizing nutritional status, as reflected by serum albumin and guided by protein intake targets, should be considered a fundamental component of comprehensive anemia management. Future prospective studies incorporating detailed dietary assessments are warranted to further elucidate the precise relationship between protein intake, albumin levels, and Hb target achievement. Furthermore, hypoalbuminemia often reflects poor nutritional intake and persistent uremic symptoms such as anorexia and nausea, which are common in patients with low dialysis frequency and inadequate dialysis adequacy. This state of chronic undernutrition and uremic burden can lead to refractory anemia that is less responsive to ESA and iron therapy, potentially increasing the need for blood transfusion. Iron status, a central factor in erythropoiesis, was also significant; lower transferrin saturation (TSAT) was a major risk factor for Hb non-attainment, consistent with DOPPS findings (28). Ogawa et al. further confirmed that maintaining TSAT ≥20% facilitates Hb improvement in MHD patients (29), supporting our conclusions. Hence, optimizing TSAT and iron utilization through tailored iron supplementation is crucial for effective anemia management and stable Hb levels. Disturbances in calcium and phosphate metabolism also play a vital role, our study found that higher serum calcium and lower phosphate levels favored Hb target attainment. The literature suggests that the mechanisms underlying this association may be multifaceted: On the one hand, calcium was required for the *in vitro* proliferation and differentiation of erythroid progenitor cells induced by EPO (30, 31). Higher serum calcium levels may promote erythropoiesis by enhancing the survival and differentiation of erythroid progenitor cells. On the other hand, hyperphosphatemia impairs erythropoiesis through multiple pathways: it can directly inhibit erythropoiesis by accumulating toxic polyamines (32); it can also indirectly impair erythropoiesis by suppressing vitamin D synthesis, leading to hypocalcemia and elevated parathyroid hormone (PTH) levels (33, 34). Furthermore, high serum phosphorus levels induce an increase in FGF-23, which in turn exacerbates vitamin D deficiency, forming a vicious cycle that sustains both hyperphosphatemia and anemia (34, 35). As an inflammation marker, elevated C-reactive protein (CRP) levels were independently associated with Hb non-attainment. Inflammation impairs erythropoiesis via multiple pathways, including suppression of erythroid progenitor proliferation, inhibition of endogenous erythropoietin production, and induction of hepcidin expression, thereby restricting iron availability (36, 37). These findings highlight the critical impact of inflammation on anemia management in MHD patients.

Based on the multicenter database of the Wuhan Hemodialysis Quality Control Center, this study identified independent factors associated with Hb target achievement in MHD patients. Nevertheless, several limitations should be acknowledged. As a retrospective analysis, the study may have overlooked unmeasured confounders such as dynamic medication dose adjustments, patient adherence, occult blood loss and subclinical myelodysplastic syndromes not detected without bone marrow examination, could have influenced the results. Second, the occurrence of blood transfusions and major bleeding events during the observation period represents a significant confounding variable in assessing the natural course of anemia management. The classification relying on a single Hb measurement may also be influenced by short-term variability. Third, the menopausal status of female patients was not recorded, which could confound the observed association between female sex and lower Hb target achievement. Furthermore, inflammation assessment was limited to C-reactive protein (CRP) levels, without including other key regulators of erythropoiesis such as interleukin-6 (IL-6) or hepcidin. The observed lower Hb attainment in patients with tunneled or non-tunneled catheters (TCC/NCC) might be attributable to catheter-related microinflammation or hemodynamic changes; however, relevant factors like catheter duration and infection history were not controlled for in this study. Additionally, although all centers adhere to the same quality control framework, potential variations in hospital level (tertiary vs. secondary) and geographic location (urban vs. rural) were not adjusted for in our analysis. These factors might affect resource availability and standard of care, potentially confounding the observed associations. To address these gaps, future prospective cohort studies with more comprehensive data collection are needed, ideally incorporating machine learning approaches to develop predictive models for Hb management. Additionally, randomized controlled trials are essential to validate these findings and further optimize anemia management strategies, ultimately improving Hb target rates in the MHD population.

## 5 Conclusion

This multicenter retrospective study, encompassing a large cohort of MHD patients in Wuhan, identified a range of modifiable and non-modifiable factors independently associated with Hb target achievement. Key risk factors for Hb non-attainment included female sex, use of tunneled cuffed catheters, lower dialysis frequency, poor pre-dialysis blood pressure control, absence of levocarnitine supplementation, higher serum phosphorus, and elevated CRP levels. In contrast, protective factors such as the use of low molecular weight heparin, higher serum albumin and calcium levels, adequate iron status (reflected by TSAT), and better healthcare coverage significantly contributed to improved anemia control. These findings underscore the multifactorial nature of renal anemia and the importance of comprehensive, individualized management strategies. Specifically, beyond the core regimen of ESAs and iron, optimizing dialysis adequacy and nutritional support is fundamental to ameliorate uremic symptoms and poor appetite, thereby addressing a key driver of refractory anemia. Further prospective and interventional studies are warranted to validate these associations and support the development of precision medicine approaches to optimize anemia outcomes in the dialysis population.

## Data Availability

The raw data supporting the conclusions of this article will be made available by the authors, without undue reservation.

## Footnotes

1
https://qc.xuetou.net.cn/qc-web/login
